# Seeing less to perform better: stroboscopic stimulation effects on padel volley accuracy and reactive agility

**DOI:** 10.7717/peerj.21038

**Published:** 2026-03-30

**Authors:** Jesus Vera, Paula Lara, Antonio Francisco Ruiz-Pérez, Teresa Zwierko, Amador García-Ramos, Beatriz Redondo

**Affiliations:** 1 New England College of Optometry, Boston, United States of America; 2 Department of Optics, Universidad de Granada, Granada, Spain; 3 Center for Structural and Functional Human Research, University of Szczecin, Szczecin, Poland; 4 Department of Sports Sciences and Physical Conditioning, Universidad Católica de la Santísima Concepción, Concepción, Chile

**Keywords:** Racquet sports, Optometry, Psychomotor performance, Sports performance

## Abstract

**Objectives:**

The integration of stroboscopic eyewear into sport-specific warm-ups has demonstrated potential for enhancing performance. This study investigated the immediate effects of stroboscopic stimulation on sport-specific performance in padel, focusing on volley accuracy and reactive agility.

**Methods:**

Twenty competitive male padel players (26.0 ± 6.3 years) completed two experimental sessions in a counterbalanced within-subjects design: one involving a warm-up with stroboscopic eyewear (5 Hz, 50% duty cycle) and another under normal visual conditions. Performance was evaluated during and after the warm-up in each condition using a volley accuracy task, and a light-based reactive agility test was assessed after the warm-up.

**Results:**

Volley accuracy performance was significantly reduced under stroboscopic conditions compared to control conditions (*p* < 0.001, *d* = 3.14). However, volley performance significantly improved after the stroboscopic warm-up compared to the control post-warm-up (*p* = 0.041, *d* = 0.52). No statistically significant differences were found for reactive agility (*p* = 0.092), though a small-to-moderate effect size favored the stroboscopic condition (*d* = 0.41).

**Conclusions:**

A single warm-up session using stroboscopic stimulation can acutely enhance volley accuracy in padel once normal vision is restored, suggesting potential for perceptual-motor priming. Effects on reactive agility were inconclusive, potentially due to task specificity and stimulus limitations. These insights may help coaches boost performance by integrating this approach into pre-training and pre-competition routines.

## Introduction

In fast-paced sports, athletes depend on split-second visual judgments to anticipate opponents’ actions and respond effectively (Laby & Appelbaum, 2021). Due to the increased recognition of visual perception in athletic performance, there has been growing interest in training methods aimed at enhancing athletes’ visual processing abilities (Lochhead et al., 2024). One such approach is perceptual-cognitive training using stroboscopic stimulation, which involves intermittently disrupting visual input to increase neural processing demands during practice (Wilkins & Appelbaum, 2020). This method has demonstrated to improve the efficiency of visual processing and motor responses under challenging conditions (Appelbaum et al., 2011; Wilkins & Gray, 2015; Ellison et al., 2020). In relation to sport-specific performance, several studies have shown that stroboscopic training improves response accuracy and reaction time across various sports disciplines (Zwierko et al., 2024a; Li et al., 2024a; Fortes et al., 2025; Hülsdünker, Gunasekara & Mierau, 2021; Wang et al., 2026; Vera et al., 2025).

Previous research has demonstrated that short-term exposure to stroboscopic visual conditions may acutely impair task performance due to the reduced availability of visual information (Beavan et al., 2021; Fransen et al., 2017; Heuvelmans et al., 2024; Li et al., 2024b; Smith et al., 2024). However, this same mechanism has been hypothesized to elicit adaptive training effects when used appropriately, with improvements often observed after the cessation of stroboscopic exposure (Wilkins & Appelbaum, 2020). In fact, there is scientific evidence suggesting that even a single session of stroboscopic exposure can lead to acute improvements in a multiple object tracking task (Bennett, Hayes & Uji, 2018) or reactive agility in soccer players (Zwierko et al., 2024b).

Padel, a rapidly growing racket sport, demands a high degree of reactivity, coordination, and precision under time-constrained conditions (Courel-Ibáñez, Martínez & Cañas, 2017). Success in padel relies not only on technical skill but also on an athlete’s ability to rapidly process visual stimuli and adapt to constantly changing game scenarios (Garcia-Gimenez et al., 2022). Due to the sport’s emphasis on close-range interactions and rapid ball exchanges, improving visual-perceptual processing may lead to significant performance gains.

Despite growing interest in this area, no research exists on how stroboscopic stimulation specifically affects padel performance, particularly in tasks directly related to sport-specific accuracy and reactive agility. The main objective of this study was to evaluate the acute effects of stroboscopic stimulation on padel-specific performance, assessing outcomes both during stroboscopic exposure and immediately after a stroboscopic warm-up. This approach allowed us to distinguish between potential performance decrements under restricted visual input and potential performance enhancements once normal vision was restored. By focusing on padel-relevant measures such as volley accuracy and reactive agility, this study seeks to provide applied insights into the potential utility of stroboscopic training in racquet sports. Based on previous research (Zwierko et al., 2024b), we hypothesized that stroboscopic stimulation would acutely modulate padel-specific performance, with volley accuracy improving immediately after the stroboscopic warm-up once normal vision was restored. We further hypothesized that reactive agility would also improve following stroboscopic exposure. However, given that this is the first study to assess reactive agility using these specific sport-specific metrics in padel, this hypothesis was considered exploratory and formulated with caution.

## Materials and Methods

### Sample size estimation and justification

The sample size was estimated *a priori* to ensure sufficient statistical power (0.80) to detect a meaningful within-subject effect of the intervention (stroboscopic *vs.* control) across two time points (during *vs.* post warm-up) using a repeated-measures ANOVA (within factors). The calculation was performed using G*Power (version 3.1). The effect size estimate was based on data from Zwierko et al. (2024b), who reported a Cohen’s d of 0.57 for the acute effects of stroboscopic exposure during warm-up on reactive agility tasks involving ball dribbling in soccer players. We used this value directly as our smallest effect size of interest. Assuming a two-tailed test, alpha = 0.05, power = 0.80, and a correlation of 0.5 among repeated measures, the required sample size was 18 participants.

### Participants

A total of 20 competitive male padel players (age: 26.0 ± 6.3 years; experience: 7.8 ± 3.3 years) participated in the study. All participants reported normal or corrected-to-normal vision and no history of neurological or musculoskeletal impairments. Written informed consent was obtained from each participant before the start of the study, and all procedures adhered to the Declaration of Helsinki and were approved by the University of Granada Institutional Review Board (1961-N-22).

### Experimental design and procedure

**Figure 1:**
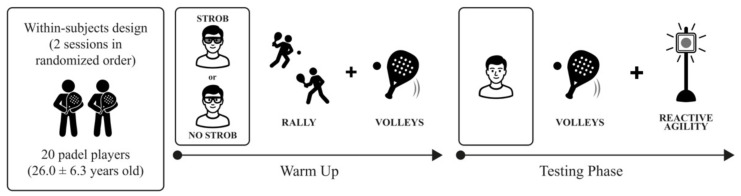
Schematic illustration of the experimental design. STROB, stroboscopic condition; NO STROB, non-stroboscopic condition (normal vision).

A within-subject, repeated-measures design was employed to evaluate the effects of stroboscopic stimulation on padel-specific performance. Each participant completed two experimental conditions: (1) stroboscopic eyewear and (2) normal vision (control). Performance was assessed both during the intervention (with stroboscopic eyewear or under normal vision) and immediately after, always under normal vision conditions (see Fig. 1 for a schematic illustration of the experimental design). All testing sessions were conducted on the same indoor padel court under comparable environmental conditions. The order of experimental conditions was fully counterbalanced across participants to minimize learning and fatigue effects.

Stroboscopic stimulation was delivered using eyewear (Senaptec Strobe, Beaverton, OR, USA) at a frequency of five Hz, corresponding to a cyclical pattern of 200 ms, with 100 ms of visibility (transparent lenses) followed by 100 ms of occlusion (opaque lenses). This mode of intermittent visual stimulation was intended to disrupt the continuous visual flow, thereby increasing perceptual load and training the athlete’s visual-motor system under constrained conditions (Wilkins & Appelbaum, 2020).

Each session began with a padel-specific warm-up lasting 15 min, performed either with or without the use of stroboscopic stimulation, depending on the condition. In the non-stroboscopic stimulation condition, participants wore the glasses (without using any stimulation) to control for the potential effects associated to its use (*e.g.*, discomfort or reduced field of view). The warm-up was designed to mimic a standard pre-match routine. It started with both players rallying from the back of the court, executing both forehand and backhand shots, including wall rebounds. After this phase, one player moved to the offensive zone (near the net) to practice forehand and backhand volleys, followed by the second player taking a turn in the same position. This ensured that all participants engaged in a representative, sport-specific warm-up protocol before task execution. After this, players performed the padel-specific accuracy test (see below) with or without stroboscopic eyewear, which was also considered as warm-up.

### Padel-specific tests

**Figure 2:**
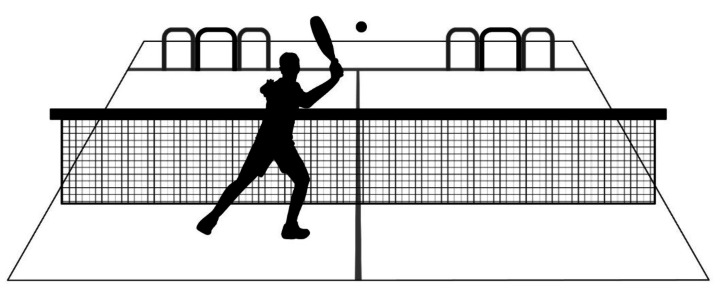
Schematic illustration of the padel-specific accuracy task. The central gates (90 cm wide × 70 cm high) provide 2 points per successful volley, while the two outer gates (70 cm wide × 70 cm high) giving 1 point each.

To assess accuracy, we designed a task in which players performed volleys to designated targets (Fig. 2). An experienced padel coach fed balls from the back end of the court, and the player was instructed to volley to the specified side (right or left) as directed by the coach (the sequence of the side order was randomly established), including forehand and backhand volleys in random order. Each condition included 50 trials, divided into two sets of 25 trials, with a 2-minute break between sets. This test was performed in the warm-up (performed with or without stroboscopic glasses according to the experimental sessions), as well as after completing the warm-up (always performed without stroboscopic glasses). As result, participants performed a total of 100 volleys per experimental condition (50 in the warm-up phase, and 50 after completing the warm-up). Successful volleys through the central gate (90  × 70 cm) were worth 2 points, and those through the outer gates (70  × 70 cm) 1 point each, yielding a maximum possible score of 100. Although ball feeding was performed manually by an experienced coach to preserve ecological validity, feeding instructions were standardized to minimize variability across trials.

**Figure 3:**
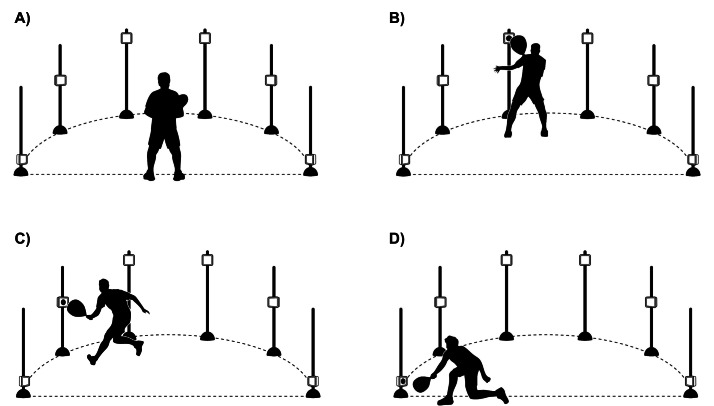
Schematic illustration of the padel-specific reactive agility task. (A) The padel player is positioned in the starting position, while (B, C, and D) show the player reaching the illuminated target with the racket. The lights are positioned 2 m from the starting point, covering a 180° field of view.

For the padel-specific reactive agility task, we designed a test using the Neural Trainer^™^ system (Neural Trainer, Montevideo, Uruguay), with six lights serving as the triggering stimulus. This reactive agility task involved a series of 30 trials in which players responded to randomly activated light targets placed 2 m from their starting position (Fig. 3). The targets, mounted on vertical poles at various heights (0.5, 1 and 1.5 m), were designed to simulate realistic on-court movement patterns. Upon the activation of each light (in red), the player had to move quickly and reach the illuminated target with their racket (triggered at 30 cm of distance) before returning to the starting position. This task was intended to assess the player’s reaction speed in applied contexts. The distribution and timing of the lights were randomized to prevent anticipation and to closely replicate the dynamic nature of competitive padel. The accumulated time required to reach the 30 lights was considered to determine reactive agility performance.

### Statistical analysis

Descriptive data are presented as means and standard deviations. A two-way repeated-measures analysis of variance (ANOVA; type of intervention (stroboscopic glasses or control) and point of measure (during warm-up and post warm-up)) was conducted to analyze padel volley accuracy performance. Bonferroni *post hoc* corrections were applied when comparing scores across the four experimental conditions. A paired-samples *t*-test was applied to the time required to complete the reactive agility task after performing a padel-specific warm-up with stroboscopic stimulation or under normal vision conditions. Because reactive agility was assessed only once per condition (post warm-up), a paired-samples *t*-test was considered the most appropriate analytical approach. The level of statistical significance was set at *p* = 0.05, and effect sizes were also reported to facilitate interpretation of the magnitude of changes. The effect sizes were interpreted according to the following criteria: small (0.2), medium (0.5), and large (0.8) for Cohen’s d, and small (0.01), medium (0.06), and large (0.14) for partial eta squared (Cohen, 1988). All analyses were conducted using the JASP statistical analysis software (version 0.19.3).

## Results

**Figure 4:**
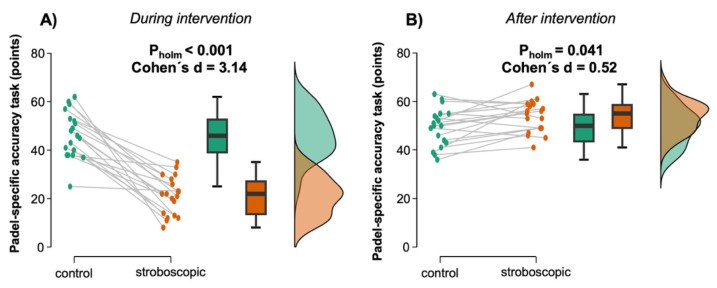
Effects on padel-specific accuracy during (A) and after (B) stroboscopic intervention. Individual data points representing each participant’s padel-specific accuracy scores, connected by gray lines to indicate within-subject changes between the control (green) and stroboscopic (orange) conditions. The box plots display the median (horizontal line), interquartile range (IQR) (box edges), and minimum/maximum values within 1.5 × IQR (whiskers) for each group. The density plots illustrate the overall distribution of scores, highlighting the spread and central tendency of the data for each condition.

Descriptive values of the padel-specific metrics assessed in the study are reported in Table 1. The ANOVA performed to assess the effects on volley accuracy revealed statistically significant differences for the main effect of *type of intervention* (*F* = 35.24, *p* < 0.001, *η*^2^_p_ = 0.66) and *point of measure* (*F* = 244.50, *p* < 0.001, *η*^2^_p_ = 0.93), as well as for the interaction *type of intervention* × *point of measure* (*F* = 89.04, *p* < 0.001, *η*^2^_p_ = 0.83). *Post-hoc* analyses showed a very large reduction in volley accuracy during stroboscopic exposure in comparison to control conditions (p-corrected < 0.001, Cohen’s *d* = 3.14) (Fig. 4A). For its part, the effects observed after the intervention revealed a moderate increase in volley accuracy after performing the warm-up with the strobe glasses in comparison to the control condition (p-corrected = 0.041, Cohen’s *d* = 0.52) (Fig. 4B).

**Figure 5:**
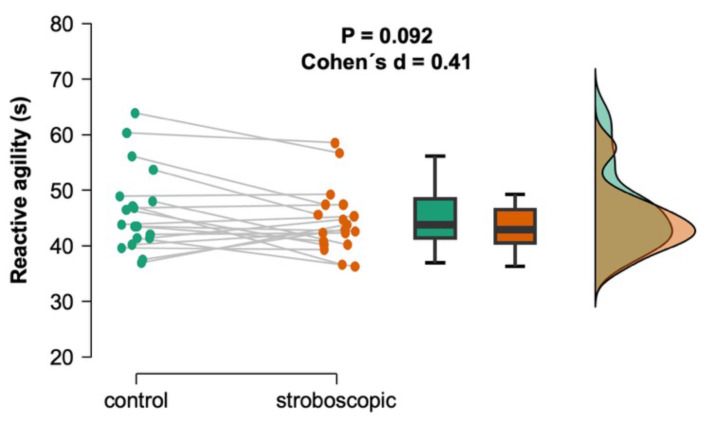
Effects on padel-specific reactive agility after stroboscopic intervention. Individual data points representing each participant’s padel-specific reactive agility, connected by gray lines to indicate within-subject changes between the control (green) and stroboscopic (orange) conditions. The box plots display the median (horizontal line), interquartile range (IQR) (box edges), and minimum/maximum values within 1.5 × IQR (whiskers) for each group. The density plots illustrate the overall distribution of scores, highlighting the spread and central tendency of the data for each condition.

The reactive agility test performed after the intervention (post warm-up) did not show statistically significant differences between the stroboscopic and control conditions (*t* = 1.78, *p* = 0.092, Cohen’s *d* = 0.41) (Fig. 5).

**Table 1:** Descriptive values for the padel-specific metrics assessed during and after the experimental intervention (stroboscopic and control).

		**Point of measure**	
	**Intervention**	*During warm-up*	*Post warm-up*
*Padel-specific accuracy task (points)*	*Control*	46.42 ± 9.56	49.47 ± 7.85
	*Stroboscopic*	21.16 ± 7.94	53.68 ± 6.57
*Padel-specific reactive agility task (s)*	*Control*	–	46.37 ± 7.47
	*Stroboscopic*	–	44.32 ± 5.82

## Discussion

The present study examined the effects of stroboscopic stimulation on padel-specific performance, focusing on volley accuracy and reactive agility. Our findings indicate that while performance in the accuracy task was substantially impaired during the use of stroboscopic eyewear, a moderate enhancement was observed immediately after the stroboscopic warm-up. Regarding reactive agility, no statistically significant differences were observed following the intervention (post warm-up), though a small-to-moderate effect size was noted in favor of the stroboscopic condition.

The substantial impairment in volley accuracy observed during stroboscopic exposure (54.4% reduction compared to the control condition) is consistent with previous research indicating that intermittent visual occlusion temporarily degrades perceptual input, thereby increasing the cognitive and motor demands during task execution (Erickson, 2020). However, it should be noted that the magnitude of this effect is highly dependent on the specific characteristics of stroboscopic stimulation, with frequency (higher frequencies being less challenging) and duty cycle (higher duty cycles being less challenging) serving as the most critical parameters (Wilkins & Appelbaum, 2020; Appelbaum & Erickson, 2018). For example, Heuvelmans et al. (2024) reported a 37.6% reduction in passing accuracy in soccer players using a stroboscopic stimulation at three Hz. This reduction in performance is thought to play a crucial role in the adaptive process, promoting long-term enhancements in neural efficiency and perceptual precision once normal visual conditions are restored (Appelbaum & Erickson, 2018).

Our data revealed that stroboscopic exposure during a padel-specific warm-up led to a significant improvement in volley accuracy immediately after the intervention, with a moderate effect size (Cohen’s *d* = 0.52). This result aligns with previous findings in perceptual–cognitive training, where brief visual perturbation was found to induce compensatory adaptations in attention and sensory prediction mechanisms once full visual input was restored (Wilkins & Appelbaum, 2020; Appelbaum & Erickson, 2018). Given that padel requires rapid information processing and precise execution in confined playing situations, the observed improvements may indicate enhanced visuomotor coordination and anticipatory skills, which are especially important during volleys where precise timing and spatial accuracy are essential. The current findings support the practical utility of including stroboscopic stimulation during warm-ups to boost visuomotor performance in dynamic racquet sports like padel. However, to the best of our knowledge, no previous studies have examined the acute effects of stroboscopic stimulation on sport-specific response accuracy.

Regarding padel-specific reactive agility, we did not observe a statistically significant improvement following the stroboscopic warm-up, although the small-to-moderate effect size (Cohen’s *d* = 0.41) suggests a possible performance benefit. This finding may be partly explained by the light-based nature of the agility task, which, despite being highly standardized, may lack sufficient ecological validity to fully capture the perceptual–motor demands of padel. A recent study conducted by Zwierko et al. (2024b) in soccer players found significant improvements in reactive agility during soccer-specific drills involving ball dribbling, whereas Strainchamps et al. (2023) did not observe significant benefits of using stroboscopic stimulation during warm-up in sport-specific reaction speed in international-level table tennis athletes. There are several factors that could partially explain the mixing findings, including the warm-up duration, type of stroboscopic stimulation, sport discipline, athletes’ expertise or type of task among others. The mediating role of the mentioned factors should be assessed in future investigations.

From a practical standpoint, these findings suggest that short-term use of stroboscopic eyewear during warm-up routines could serve as an effective priming tool to enhance accuracy in padel. Specifically, the increase in volley accuracy after warm-up in stroboscopic conditions was 15.6%, whereas this improvement was 6.6% in normal vision conditions. These percentage gains represent a meaningful competitive advantage in sport contexts, where subtle differences can determine the difference between winning and losing at professional levels. Coaches and practitioners may consider incorporating stroboscopic stimulation in pre-competition or training environments to temporarily boost players’ focus and visuomotor readiness, as it has been proposed for mental fatigue (Díaz-García et al., 2024). A key advantage of this vision training approach, categorized as naturalistic training (Appelbaum & Erickson, 2018), is its ability to be implemented in sport-specific contexts, thereby maximizing transfer to real-world performance scenarios. Both the drills and the stroboscopic parameters (*e.g.*, frequency and duty cycle) must be carefully adapted to the specific sport discipline and athlete’s characteristics to optimize progression and account for learning effects. For example, less challenging stroboscopic settings (*i.e.,* higher frequencies and longer duty cycles) may be appropriate for initial exposure or less experienced athletes, whereas more demanding configurations (lower frequencies and shorter duty cycles) can be progressively introduced in advanced players to increase perceptual load without excessively impairing performance.

The findings of this study contribute to the growing literature on the applicability of perceptual-cognitive training tools in sport-specific contexts. To our knowledge, this is the first investigation assessing the immediate effects of stroboscopic stimulation on padel performance, providing novel insights into how this strategy could be implemented for enhancing padel performance. Nonetheless, this study is not exempt of limitations, and they must be acknowledged. First, the experimental sample comprised only male intermediate-to-advanced players, limiting the generalizability to other groups (*i.e.,* female or amateur players) who may present different perceptual-motor adaptations. Second, padel has specific characteristics, and the external validity of the current results to other sport disciplines requires further investigation. Third, in evaluating reactive agility, we used a non-specific (light-based) stimulus. While this type of stimulus offers the advantage of being easily standardized and repeatable across trials, it may lack ecological validity (Paul, Gabbett & Nassis, 2016). Previous research suggests that tests using specific, human-based stimuli may better reflect real-game scenarios (Morral-Yepes et al., 2022). Lastly, the intervention was acute, and its impact was examined immediately after the experimental manipulation. Further research is warranted to examine the effects of longer training programs, retention over time, and transfer to competitive match play, as well as to identify the optimal range of performance decrement induced by stroboscopic exposure that may support the individualized prescription of stroboscopic training in sport-specific contexts.

## Conclusions

Our findings support the short-term benefits of incorporating stroboscopic warm-up routines to enhance volley accuracy in padel. Although the benefits for reactive agility remain inconclusive, the observed trends suggest potential for perceptual-motor priming effects. Overall, stroboscopic stimulation represents a promising and ecologically valid approach to acutely enhance visuomotor readiness in dynamic sports. Future investigations should examine the effects of task characteristics, stimulus specificity, intervention duration, and stroboscopic stimulation parameters (*i.e.,* frequency and duty cycle) on performance outcomes.

##  Supplemental Information

10.7717/peerj.21038/supp-1 Supplemental Information 1 Raw data of the different dependent variables

## Additional Information and Declarations

### Data Availability

The following information was supplied regarding data availability: The raw data is available in the Supplemental File.
